# Ultrasonic Dissection versus Conventional Dissection for Pancreatic Surgery: A Meta-Analysis

**DOI:** 10.1155/2016/6195426

**Published:** 2016-01-10

**Authors:** Haiming Lei, Dong Xu, Xinghua Shi, Koulan Han

**Affiliations:** ^1^School of Clinical Medicine, Yancheng Institute of Health Sciences, Yancheng 224005, China; ^2^Department of General Surgery, Gaochun People's Hospital, Nanjing 211300, China

## Abstract

*Background*. The role of ultrasonic dissection (UD) in pancreatic surgery remains controversial. The aim of this meta-analysis was to evaluate the clinical effect of UD in pancreatic surgery when compared with conventional dissection (CD). *Materials and Methods*. A comprehensive literature search was performed to identify eligible studies that compared UD with CD for pancreatic surgery in PubMed, EMBASE, Web of Science, and the Cochrane Library. Risk ratio (RR) or mean difference with 95% confidence interval (CI) was calculated. *Results*. Six studies were included with a total of 215 patients undergoing UD and 210 undergoing CD. In comparison with CD in distal pancreatectomy, UD was associated with lower rates of pancreatic fistula (RR = 0.46, 95% CI: 0.27–0.76) and abdominal abscess and shorter operation time and hospital stay (*P* < 0.05). In pancreaticoduodenectomy, there was no significant difference in pancreatic fistula rate between two groups (RR = 0.79, 95% CI: 0.48–1.29). However, the significantly less intraoperative blood loss and the transfused blood unit were found in patients receiving UD (*P* < 0.05). *Conclusions*. The results of this meta-analysis show that, in comparison with CD, UD is associated with better perioperative outcomes in pancreatic surgery.

## 1. Introduction

Pancreatic resection remains the effective treatment for both benign and malignant pathologies of the pancreas and the surroundings. The procedures include pancreaticoduodenectomy (PD), pylorus-preserving PD (PPPD), and distal pancreatectomy (DP). All these procedures require time-consuming, extensive tissue and vessel dissection with a high risk of increasing the quantity of intraoperative blood loss and the number of transfused blood units. Although the operative mortality rate has markedly declined to <5% with increasing experiences and advances in medical and surgical technology [1–3], the postoperative morbidity rate remains about 40% [4, 5]. The most common and serious complication is pancreatic fistula, which can further cause abdominal abscess and hemorrhage, increase mortality rate, and prolong hospital stay [6, 7]. Therefore, it is necessary to improve the current surgical approach to achieve more satisfying perioperative outcomes.

Ultrasonic dissection (UD) device, delivering high-frequency mechanical vibration onto the targeted tissue, denatures the proteins by disrupting the hydrogen bonds within the protein structure [8, 9]. Compared to conventional dissection (CD) that involves the application of electrocautery, clips, or ligatures, UD can quickly and simultaneously cut and coagulate the tissues with significantly less damage to the adjacent tissues. Due to this advantage, the device has been widely employed in the field of laparoscopic surgery. Many studies have reported the safety and usefulness of UD device in open surgical procedures including hemorrhoidectomy [10], thyroidectomy [11], and gastric [12], colorectal [13], and hepatic surgery [14]. The application of UD device has also been investigated in pancreatic surgery [15–20]. However, the results have reached no consensus.

Thus, the aim of the present meta-analysis was to assess whether the use of UD in pancreatic surgery has clinical efficacy in improving perioperative outcomes when compared with CD.

## 2. Methods

### 2.1. Literature Search

A comprehensive literature search was conducted in PubMed, EMBASE, Web of Science, and the Cochrane Library to identify eligible studies. The following MeSH terms and text words were used in combination with Boolean operators AND or OR without language or geographical restrictions: “ultrasonics,” “ultrasonic dissection,” “harmonic scalpel,” “pancreatic surgery,” “pancreaticoduodenectomy,” and “pancreatectomy.” Reference lists of all relevant studies were screened to detect additional publications. Two of the investigators (Haiming Lei and Dong Xu) independently reviewed the titles and abstracts identified in the search. The latest search was conducted on June 1, 2015.

### 2.2. Study Selection

All published randomized controlled trials (RCTs) and non-RCTs that compared UD versus CD for pancreatic surgery were included. In case of duplicates, only the latest or the most detailed and informative article, or the one with the best quality in methodology, was selected, unless they were reports from different time periods or the data of overlapping patients could be subtracted. The exclusion criteria were in vitro experiments, animal studies, or studies with cointerventions. Case reports, reviews, letters, and conference abstracts which provided insufficient information were also excluded. Studies were considered for meeting the inclusion criteria by two reviewers (Haiming Lei and Dong Xu) with any disagreements resolved by discussion or arbitration by a third reviewer (Xinghua Shi). Cohen's kappa statistic was used to evaluate the chance-corrected agreement between reviewers (SPSS, version 18.0) [21].

### 2.3. Data Extraction

The following information regarding each eligible study was extracted using standardized data extraction forms: authors' names, year of publication, country, study design, study interval, patients' mean ages, cases per arm, type of surgery, and ultrasonic device used in each study. The outcomes of this meta-analysis were pancreatic fistula, abdominal abscess, postoperative hemorrhage, operation time, intraoperative blood loss, number of transfused blood units, postoperative hospital stay, and overall mortality and morbidity. Pancreatic fistula was defined according to the International Study Group on Pancreatic Fistula (ISGPF) [22].

### 2.4. Quality Assessment

Methodological quality of the eligible RCTs was assessed using the Jadad scoring system [23] and that of the non-RCTs was assessed by the Methodological Index for Nonrandomized Studies (MINORS) [24].

### 2.5. Statistical Analysis

This study was conducted in accordance with the Statement of Preferred Reporting Items for Systematic Reviews and Meta-Analyses [25]. The data analysis was performed using the statistical software Review Manage, version 5.1.0 (The Cochrane Collaboration, 2011). For dichotomous variables, the risk ratio (RR) for each study was aggregated in Mantel-Haenszel method to obtain a pooled RR with a corresponding 95% confidence interval (CI). For continuous variables, mean difference (MD) with 95% CI was calculated using the inverse variance method. If studies reported continuous data as median and/or range values, the standard deviation was calculated using statistical algorithms by Hozo et al. [26]. Statistical heterogeneity was assessed using Cochran's *Q* test with *P* < 0.1 considered as statistically significant. *I* ^2^ statistic was used to evaluate the impact of heterogeneity on the meta-analysis. *I* ^2^ values less than 25%, between 25% and 50%, and greater than 50% were defined as low, moderate, and high statistical heterogeneity, respectively. If the heterogeneity across studies approached statistical significance, the random effects model would be used; otherwise, the fixed effect model would be chosen. Funnel plot was constructed to detect the possibility of publication bias [27]. A symmetrical inverted funnel in the plot indicates the absence of this possibility, whereas an asymmetrical shape represents the presence of publication bias. The visual asymmetry of the funnel plot was tested by Egger's regression with *P* < 0.1 considered as significant [28].

## 3. Results

### 3.1. Study Selection

**Figure 1:**
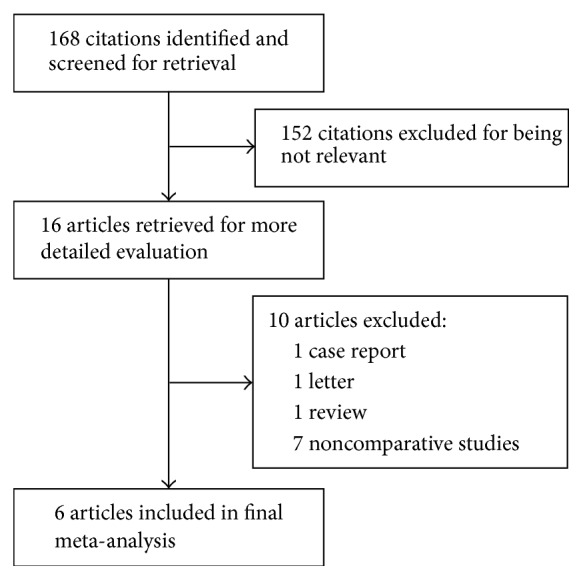
Flow chart of search.

The initial search returned 168 potentially relevant citations. After screening of titles and abstracts, 152 citations were excluded for no relevance. Of the remaining 16 articles, 10 were excluded for not meeting the inclusion criteria. Ultimately, six studies including two RCTs [15, 18] and four non-RCTs [16, 17, 19, 20] matched the criteria for inclusion. A flow diagram shown in Figure 1 details the selection process. There was excellent agreement between reviewers for study inclusion (*κ* = 0.99).

### 3.2. Study Characteristics

The major characteristics of the included studies, along with the quality assessment scores, are presented in Table 1. A total of 425 patients were included in the analysis with 215 (51%) undergoing UD and 210 (49%) undergoing CD. All of the studies were published in English-language journals between 1999 and 2014. Four studies [15–17, 20] were performed in Japan, one [19] was performed in Germany, and one [18] was performed in Germany, Italy, and Greece. The patients' mean age ranged from 56.7 years to 72 years. The sample size in each study varied from a minimum of 26 to a maximum of 109. DP procedure was performed in three studies, while PD and/or PPPD procedures were selected in the other three recent trials. Definition of pancreatic fistula was described in all included studies.

### 3.3. Meta-Analysis

#### 3.3.1. UD versus CD for DP

**Figure 2:**
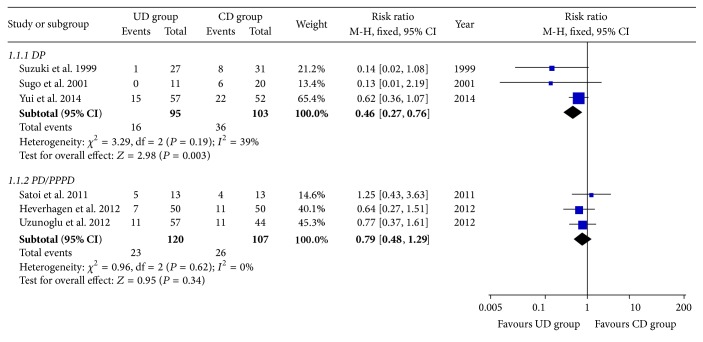
Meta-analyses of pancreatic fistula for DP and PD/PPPD.

Three studies [15, 16, 20] described the data regarding pancreatic fistula following DP. In a comparison of 95 patients undergoing UD with 103 patients undergoing CD, there was a significantly decreased risk of pancreatic fistula for UD (16.8% versus 35.0%; RR = 0.46, 95% CI: 0.27–0.76, *P* = 0.003). No significant heterogeneity among studies was found (*P* = 0.19, *I* ^2^ = 39%) (Table 2 and Figure 2). The occurrence of abdominal abscess was significantly lower in patients receiving UD than in patients receiving CD (3.2% versus 15.5%; RR = 0.24, 95% CI: 0.08–0.71, *P* = 0.01). In addition, the operation time (MD = −63.00 min, 95% CI: −116.41 to −9.59, *P* = 0.02) and the hospital stay (MD = −9.00 days, 95% CI: −17.78 to −0.22, *P* = 0.04) were significantly shorter in UD group. No significant difference was observed between two groups in terms of postoperative hemorrhage, intraoperative blood loss, overall postoperative mortality, or morbidity (Table 2).

#### 3.3.2. UD versus CD for PD/PPPD

Three studies [17–19] reported the incidence of pancreatic fistula after PD/PPPD. Overall, 23 of 120 (19.2%) patients in UD group and 26 of 107 (24.3%) patients in CD group experienced pancreatic fistula. Meta-analysis showed no significant difference in the pancreatic fistula rate between two groups (RR = 0.79, 95% CI: 0.48–1.29, *P* = 0.34) with no significant heterogeneity across studies (*P* = 0.62, *I* ^2^ = 0%) (Table 2 and Figure 2). Our meta-analysis also showed that there was no statistically significant difference between two groups in abdominal abscess rate, postoperative hemorrhage rate, operation time, hospital stay, overall postoperative mortality, or morbidity. However, the intraoperative blood loss (MD = −183.08 mL, 95% CI: −346.01 to −20.16, *P* = 0.03) and the transfused blood unit (MD = −0.69, 95% CI: −1.28 to −0.09, *P* = 0.02) were significantly less in UD group than in CD group (Table 2).

### 3.4. Publication Bias

**Figure 3:**
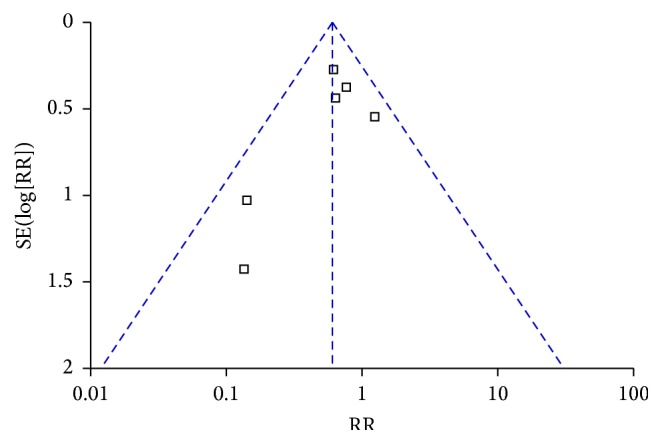
Funnel plot for publication bias.

The funnel plot, based on the incidence of pancreatic fistula, revealed visual asymmetry (Figure 3). However, the result from Egger's regression failed to show the statistical significance of such asymmetry (95% CI of intercept −4.84 to 1.14, *P* = 0.143).

## 4. Discussion

To our knowledge, this is the first meta-analysis to date that evaluates the clinical efficacy of UD in improving perioperative outcomes in pancreatic surgery when compared with CD. Our meta-analysis suggests that, in comparison with CD, UD is associated with reduced pancreatic fistula, abdominal abscess, operation time, and hospital stay following DP and related with less intraoperative blood loss and transfused blood unit after PD/PPPD. There was no difference between two groups in overall mortality or morbidity after pancreatic surgery.

Pancreatic fistula is the most common and serious complication after pancreatic resection and can further cause other complications [6, 7]. To address this intractable problem, several intraoperative measures have been investigated. Takao et al. conducted a noncomparative clinical study utilizing harmonic scalpel to transect the pancreas in biliary-pancreatic surgery [29]. They observed that there was no occurrence of pancreatic fistula in 41 patients who received reconstruction of the remnant pancreas. Suzuki et al. reported a randomized clinical trial comparing UD with CD for DP [15]. In that trial, the pancreatic stump was left open without suturing in UD group, while in CD group the stump was oversewn with mattress sutures. The trial found that pancreatic fistula rate was reduced significantly by UD (3.7% versus 25.8%). Similar result was also examined in the study reported by Sugo et al. [16]. Our meta-analysis was in concordance with these results.

Nevertheless, such advantage of UD in decreasing fistula rate was only remained in our analysis of DP procedure. The analysis of PD/PPPD did not show any difference of fistula rate between two groups. It has to be mentioned that in our analysis the devices used to cut the pancreatic parenchyma in UD groups were not consistent. For DP, the UD device was applied to transect the parenchyma in all three studies [15, 16, 20]. For PD/PPPD, however, one study [17] used UD device, while the other two [18, 19] used surgical scalpel in their UD groups for pancreas transection. Using a steel scalpel to cut the pancreas may cause abundant bleeding from the cut surface, and satisfying hemostasis will be achieved by clamping the pancreas, silk sutures, or electrocautery [29]. Clamping for hemostasis and the use of sutures may cause internal lacerations and ischemic damage of the pancreatic parenchyma [16]. However, this deleterious consequence may be avoided by the use of UD device, because a dry pancreatic stump after transection can be obtained by either the dissecting effect [15] or the sealing effect [16, 20] of UD device, and suture closure of the stump will be unnecessary. Moreover, the local temperature reached by the UD is much lower than that reached by the electrocautery. This will induce a minimal lateral energy spread and thermal injury, which is beneficial to prevent pancreatic leakage [18]. These, plus the inconsistent criteria of fistula, might be the reasons for different outcomes of pancreatic fistula rate in two types of surgery in this analysis. Accordingly, the occurrence of abdominal abscess, an indirect consequence of fistula, was decreased following pancreatic transection with UD. At the same time, the duration of hospital stay was affected and shortened in patients receiving pancreatic transection with UD due to a better postoperative course. Regrettably, the present meta-analysis did not show any difference of overall mortality or morbidity between groups. Given a much larger sample size, the potential difference of these outcomes might be detected.

Many studies have reported the superiority of UD in reducing operation time and intraoperative blood loss as compared with CD. Inoue et al. showed in a prospective randomized study that ultrasonic scalpel could significantly shorten operation time and decrease intraoperative blood loss for open gastric cancer surgery [12]. One RCT conducted by Sista et al. reported that the use of a harmonic scalpel was associated with reduced operation time and less blood loss in right colon surgery [13]. Using the UD device in axillary dissection also decreased blood loss in comparison with CD [30]. The present study demonstrated the superiority of UD in reducing bleeding during PD/PPPD, which reflects the advantage of coagulation-cutting effect of UD device. As a result, the number of transfused blood units in UD group was reduced correspondingly following PD/PPPD. However, our meta-analysis failed to show a reduction of operation time, indicating that UD is as time-consuming as CD in PD/PPPD.

Although the UD device is disposable and may potentially increase the costs of surgery, the savings of blood transfusion and suture material in pancreatic surgery might have compensated the extra costs of the UD device [17]. Moreover, Uzunoglu et al. calculated the costs of surgery in UD group and CD group and found no significant differences between two groups [18]. Besides, there is a potential advantage of UD device which is the fact that the decreased number of surgical sutures may result in a reduced incidence of surgical site infection [17].

There are some limitations in the present meta-analysis. First, we realized that inclusion of non-RCTs could not avoid an inherent selection bias in the treatment groups and may exaggerate the effect magnitude of an intervention. However, the number of RCTs comparing UD with CD for pancreatic surgery is really limited. We had to pool data from non-RCTs to reach a relatively larger sample size for evaluation of the interested outcomes in this analysis. For example, the data regarding operation time, blood loss, and hospital stay was reported by only one RCT. However, the number of such studies was increased to four and the overall sample size was turned to be nearly threefold when non-RCTs were considered. Second, clinical heterogeneity across studies was noted, which is common to all meta-analytic studies. Types of UD device, the experience of surgeons, and the slightly different definition of outcome might have influenced the results of this study, although statistical heterogeneity was not significant in all outcomes except one. Third, the publication bias may be presented in our meta-analysis due to the visual asymmetry of the funnel plot. However, we searched several databases according to the standards of the Cochrane Collaboration. Moreover, Egger's regression did not reveal any evidences for the presence of such bias. Despite these, the results of our study should be interpreted with caution.

## 5. Conclusions

The present meta-analysis shows that, in comparison with CD, UD is associated with better perioperative outcomes in pancreatic surgery, especially in DP procedure. Due to the limited RCTs in this study, future larger randomized trials are necessary for reevaluation of the clinical outcomes of the UD in pancreatic surgery.

## Figures and Tables

**Table 1:** Characteristics of the included studies.

Author	Year	Country	Study design	Study interval	Age (years)		Cases		Type of surgery	Ultrasonic device	Definition of pancreatic fistula	Quality score^*∗*^
					UD	CD	UD (M/F)	CD (M/F)				
Suzuki et al. [15]	1999	Japan	RCT	1994–1998	57.7	58.5	27 (18/9)	31 (18/13)	DP	CUSA	An external discharge of pancreatic fluid (>7 d) with an amylase level of >3 times the serum level	2/5

Sugo et al. [16]	2001	Japan	Retro.	1994–2000	61.7	56.7	11 (6/5)	20 (12/8)	DP	HS	An external discharge of pancreatic fluid (>7 d) or a high level of amylase in the drainage fluid (>3 times the serum level) on day 7 after surgery	15/24

Satoi et al. [17]	2011	Japan	PNR	2009-2010	72	64	13 (8/5)	13 (10/3)	PD, PPPD	UCS	ISGPF definition	15/24

Uzunoglu et al. [18]	2012	Germany, Italy, Greece	RCT	2009–2011	64.8	65.2	57 (33/24)	44 (29/15)	PD, PPPD	HS	ISGPF definition	3/5

Heverhagen et al. [19]	2012	Germany	PNR	2005–2011	61	64.1	50 (37/13)	50 (34/16)	PPPD	HS	ISGPF definition	16/24

Yui et al. [20]	2014	Japan	Retro.	2000–2010	66	65	57 (31/26)	52 (29/23)	DP	USAD	UD group: ISGPF definition CD group: ISGPF definition and other definitions that were not available	15/24

UD: ultrasonic dissection, CD: conventional dissection, M/F: male/female, RCT: randomized controlled trial, DP: distal pancreatectomy, CUSA: Cavitron Ultrasonic Surgical Aspirator, Retro.: retrospective observational study, HS: harmonic scalpel, PNR: prospective nonrandomized observational study, PD: pancreaticoduodenectomy, PPPD: pylorus-preserving PD, UCS: ultrasonically curved shear, ISGPF: International Study Group on Pancreatic Fistula, USAD: ultrasonically activated device.

^*∗*^Jadad score for RCTs and MINORS score for non-RCTs.

**Table 2:** Summary of meta-analysis.

Outcome	Number of studies	Number of participants		Heterogeneity	Overall effect size	95% CI of overall effect	*P*
		UD	CD				
*UD versus CD for DP*							
Pancreatic fistula	3	95	103	*P* = 0.19, *I* ^2^ = 39%	RR = 0.46	0.27–0.76	0.003
Abdominal abscess	3	95	103	*P* = 0.78, *I* ^2^ = 0%	RR = 0.24	0.08–0.71	0.01
Postoperative hemorrhage	2	68	72	NA	RR = 0.58	0.03–13.22	0.73
Operation time (min)	1	57	52	NA	MD = −63.00	−116.41 to −9.59	0.02
Intraoperative blood loss (mL)	1	57	52	NA	MD = −215.00	−695.96 to 265.96	0.38
Hospital stay (days)	1	57	52	NA	MD = −9.00	−17.78 to −0.22	0.04
Mortality	3	95	103	NA	RR = 0.91	0.06–14.22	0.95
Morbidity	1	57	52	NA	RR = 0.81	0.55–1.20	0.30
*UD versus CD for PD/PPPD*							
Pancreatic fistula	3	120	107	*P* = 0.62, *I* ^2^ = 0%	RR = 0.79	0.48–1.29	0.34
Abdominal abscess	1	13	13	NA	RR = 0.33	0.01–7.50	0.49
Postoperative hemorrhage	2	70	57	NA	RR = 1.80	0.49–6.57	0.37
Operation time (min)	3	120	107	*P* = 0.34, *I* ^2^ = 8%	MD = −5.98	−31.98 to 20.03	0.65
Intraoperative blood loss (mL)	3	120	107	*P* = 0.07, *I* ^2^ = 63%	MD = −183.08	−346.01 to −20.16	0.03
Transfused blood unit	2	107	94	*P* = 0.22, *I* ^2^ = 33%	MD = −0.69	−1.28 to −0.09	0.02
Hospital stay (days)	3	120	107	*P* = 0.58, *I* ^2^ = 0%	MD = 1.04	−2.92 to 5.00	0.61
Mortality	3	120	107	*P* = 0.66, *I* ^2^ = 0%	RR = 0.29	0.08–1.08	0.07
Morbidity	2	70	57	*P* = 0.39, *I* ^2^ = 0%	RR = 1.00	0.72–1.38	0.99

CI: confidence interval, UD: ultrasonic dissection, CD: conventional dissection, RR: risk ratio, DP: distal pancreatectomy, PD: pancreaticoduodenectomy, PPPD: pylorus-preserving PD, NA: not applicable, MD: mean difference.
